# Endoplasmic reticulum stress is involved in spiral ganglion neuron apoptosis following chronic kanamycin-induced deafness

**DOI:** 10.1042/BSR20181749

**Published:** 2019-02-08

**Authors:** Yaqin Tu, Guorun Fan, Haiying Sun, Xiong Cai, Wen Kong

**Affiliations:** 1 Department of Otorhinolaryngology, Union Hospital, Tongji Medical College, Huazhong University of Science and Technology, Wuhan 430022, China; 2 Department of Hepatobiliary Surgery, Union Hospital, Tongji Medical College, Huazhong University of Science and Technology, Wuhan 430022, China2; 3 Department of Endocrinology, Union Hospital, Tongji Medical College, Huazhong University of Science and Technology, Wuhan 430022, China

**Keywords:** Apoptosis, degeneration process, endoplasmic reticulum stress, spiral ganglion neurons

## Abstract

Aminoglycoside antibiotics-induced hearing loss is a common sensorineural impairment. Spiral ganglion neurons (SGNs) are first-order neurons of the auditory pathway and are critical for the maintenance of normal hearing. In the present study, we investigated the time-course of morphological changes and the degeneration process of spiral ganglion cells (SGCs) following chronic kanamycin-induced deafness and determined whether the endoplasmic reticulum (ER) stress was involved in the degeneration of SGNs. We detected density changes in SGCs and the expressions of Bip, inositol requirement 1 (IRE1)α, activating transcription factor-6α, p-PERK, p-eIF2α, CHOP, and caspase-12 at each time point after kanamycin treatment. Terminal deoxynucleotidyl transferase-mediated dUTP nick end labeling (TUNEL) staining was also performed. The number of SGC deletions reached ∼50% at the 70th day after kanamycin administration and the ER of most SGCs were dilated. The expression of p-PERK, p-eIF2α, p-IRE1α, Bip, caspase-12, and Chop was significantly unregulated after kanamycin treatment. The number of SGCs that were positive for both TUNEL and caspase-12 increased from day 7 to 28. Taken together, these data demonstrate that ER stress was involved in kanamycin-induced apoptosis of SGNs. Kanamycin-induced SGN apoptosis is mediated, at least in part, by ER stress-induced upregulation of CHOP and caspase-12.

## Introduction

Aminoglycoside antibiotics-induced hearing loss is a common sensorineural impairment. Spiral ganglion neurons (SGNs) are first-order neurons of the auditory pathway and are critical for the maintenance of normal hearing [1,2]. Intra-cochlear survival of ganglion cells is thought to be crucial for the successful use of cochlear implants. A cochlear implant can restore hearing function in people with severe sensorineural hearing loss (SNHL) by electrically stimulating SGNs [3]. However, Bichler et al. found that SGN death in rats deafened with aminoglycoside was sequential [4]. Thus, successful cochlear implantation depends on preventing or attenuating spiral ganglion cells (SGCs) degeneration after SNHL. To develop protective strategies for preventing SGC death, the mechanism responsible for SGC degeneration needs to be better understood. Previous studies have focused on deafness induced by a single dose of kanamycin in combination with furosemide or ethacrynic acid in guinea pigs and chronic kanamycin-induced deafness in neonatal rats [5–9]. However, there are few studies of chronic kanamycin-induced deafness in adult rats.

The endoplasmic reticulum (ER) is an organelle in which membrane-bound proteins are folded into their final advanced structures, lipids and sterols are synthesized, and free calcium is stored. Perturbation of ER function can lead to ER stress. Under ER stress, three major signal transduction proteins are triggered, i.e. PERK (interferon-induced double stranded RNA-activated protein kinase (PRKR) -like endoplasmic reticulum kinase), ATF6 (activating transcription factor 6), and IRE1 (inositol requirement 1) [10,11]. Recent studies identified the ER as an important subcellular compartment in the initiation of apoptosis. Oishi et al. found that during the early stage of aminoglycoside treatment, the function of the ER is affected, implying that these organelles play a crucial role in the initial phase of aminoglycoside-induced outer hair cell degeneration [12]. However, whether kanamycin treatment can induce ER stress directly in SGNs and whether ER stress is involved in SGN apoptosis remain unclear.

Here, we investigated the time sequence of morphological changes in the SGCs of adult rats following chronic kanamycin-induced deafness. The densities changes in SGCs were quantified. The expression levels of Bip, IRE1α, phospho-eIF2-α, phospho-PERK, CHOP, and ATF-6α at each time-point after kanamycin treatment were investigated to explore whether the function of the ER was affected. Meanwhile, the expression of caspase-12 was also measured.

## Materials and methods

### Animals and deafening procedure

All experiments in this study were carried out in strict accordance with the recommendations in the Guide for the Care and Use of Laboratory Animals of the National Institutes of Health. The protocol was approved by the Committee on the Ethics of Animal Experiments of the University of Huazhong University of Science and Technology. Ninety-six male Sprague-Dawley rats (initial body weight 125–150 ***g***, 5–6 weeks old) were obtained from the experimental animal center of Tongji Medical College, Huazhong University of Science and Technology, and used for the experiments. The animals had free access to water and food, and were allowed 1 week of acclimation before the first treatment. The animals were divided randomly into one control group and seven experimental groups. The control group (*n*=12) was treated with an equal volume of 0.9% saline for 10 days as those in the experimental groups instead of kanamycin. Experimental groups (*n*=12 for each group: 1, 7, 14, 28, 56, 70, and 140 days after kanamycin treatment) received 500 mg of kanamycin sulfate/kg bodyweight per day by subcutaneous injection for 10 days synchronously. The animals were weighed every day and the injection dosage was adjusted accordingly.

### Evaluation of auditory function

Auditory thresholds were tested by evoked auditory brain stem responses (ABRs). The tests were taken twice for each animal, first prior to the beginning of administration and then at different time after kanamycin treatment. The ABRs were measured essentially as described in the previous study [13]. The rats were anesthetized with an intraperitoneal injection of 100 mg of ketamine and 5 mg of chlorpromazine/kg body weight and kept warm with a heating pad. The ground electrode was subdermally inserted at the vertex, in the midline of the scalp between the external auditory canals. An active needle electrode was placed below the pinna of one ear and the reference electrode was inserted contralaterally. Tone bursts of 4, 8, 16, 24, and 32 kHz (10 ms duration, 1 ms rise/fall time) were generated using a SigGen software package (Tucker-Davis Technologies, Gainsville, FL, USA) and delivered to one external auditory meatus in a closed acoustic system through an ear bar connected to a EC1 Electrostatic Speaker (Tucker-Davis Technologies, Gainsville, FL, USA). The average responses to 512 stimuli were obtained for each frequency by reducing the sound intensity in 10 dB steps and finally at 5 dB intervals near threshold as described below. Intensities that appeared to be near threshold were repeated. Threshold was defined as the lowest intensity at which a reproducible response was seen that contained at least two peaks and had amplitudes of at least 0.5 μV. ABR data were collected from both ears in all rats. The final ABR of each animal was taken and interpreted by an observer blinded to the given treatment.

### Tissue preparation

At different time points (1, 7, 14, 28, 56, 70, and 140 days) after kanamycin treatment, three rats were anesthetized with ketamine/chlorpromazine as described above. The rats were transcardially perfused with normal saline, followed by 4% paraformaldehyde in 0.1 M phosphate-buffered saline (PBS), pH 7.4. After decapitation, the temporal bones were dissected. Both cochleae were isolated and the oval and round windows were opened. Cochleae were placed in the same fixative for 24 h at 4°C and decalcified with 10% EDTA in 0.1 M phosphate buffer. Following decalcification, the cochleae were rinsed with distilled water, dehydrated with a graded alcohol series, cleared in xylene and embedded in paraffin wax. Cochleae were sectioned serially at 5 μm thick parallel to the modiolus and mounted on slides. Every fifth midmodiolar section was collected for SGN density measurement after hematoxylin and eosin staining༌and five from the collected sections were randomly chosen for statistical analysis for each rat. And the remaining midmodiolar sections were used for immunohistochemical and terminal deoxynucleotidyl transferase-mediated dUTP nick end labeling (TUNEL) staining as described below.

### SGN density measurements

The time course of SGN loss in the cochlear basal turn was identified. Cell counts were performed in a single-blinded manner. Every fifth section was selected for SGC counts to eliminate the possibility of repeated counting. After hematoxylin and eosin staining, sections were examined using an Olympus microscope (1X71, Tokyo, Japan) equipped with an Olympus color video camera. Both the type-I and type-II surviving SGCs with unique visible nuclei and nucleoli were all counted in each selected section by using Image-Pro Plus 6.0 software (Media Cybernetics, Inc., Bethesda, MD, USA). SGC density was calculated by dividing the number of SGCs by the cross-sectional area of Rosenthal’s canal and expressed as the mean number of SGCs per mm^2^, as described in the literature [14]. The images were adjusted using Adobe Photoshop 8.0 software (Adobe Systems, Mountain View, CA, USA).

### Transmission electron microscopy

For electron microscopy studies, three animals were decapitated quickly after deeply anaesthetized and the bilateral cochleae were isolated. One lateral cochlea for each animal was fixed in 2.5% glutaraldehyde phosphate immediately for observing the ultrastructure. The other cochlea was stored at −80°C for western blot. After the opening of oval and round windows, the cochleae were placed in the same fixative for 24h at 4°C and decalcified with 10% EDTA in 0.1 M phosphate buffer. Following decalcification, the cochleae were rinsed with PBS and quarter turns of the cochlea were dissociated delicately. The cochlear tissues were post-fixed for 2 h in 1% osmium tetroxide, dehydrated with an ascending graded alcohol and acetone series, immersed in acetone and Epon 812 for 2 h and in Epon 812 for 2 h, and embedded in Epon 812 for 10 h at 80°C, as described previously [15]. In order to investigate the ultrastructural changes of the SGCs morphology, ultrathin sections (50 nm) of the basal turn of the cochlea were prepared with ultramicrotomy (Leica Ultracut UTC, Germany) with a glass knife, mounted on 300 mesh copper grids, contrast-stained with uranyl acetate and Lead Citrate, and examined with a Transmission Electron Microscope (FEI Tecnai G212, Phillips, Holland). The morphology of the SGCs was studied in the basal turn of the cochlea as representative, because the effect of aminoglycoside on SGC density of the basal turn is regarded as most prominent. Furthermore, there is a considerable intracochlear regional variation in perikaryal area after aminoglycoside treatment. Therefore, SGCs located at the basal turn were examined and photographed for ultrastructural analysis.

### Immunohistochemistry

Following dewaxed, slides were washed in (PBS; pH 7.4). Then the slides were placed in a Coplin jar containing citric acid solustion (pH 6.0), boiled in microwave oven for 10 min for antigen retrieval and cooled at room temperature. Slides were subsequently rinsed, incubated in 1.5% normal goat serum for 1 h at room temperature, and then placed in rabbit anti-rat caspase-12 primary antibody at a dilution of 1:200 overnight at 4°C. The slides were rinsed again, followed by incubation with Cy3-conjugated secondary antibody for 1 h at room temperature. Rinsed again, the slides were counterstained with DAPI. After immunolabeling, the sections were then processed with TUNEL staining as described below. Control sections were incubated with the diluent of primary antibody to test the specificity of the immunoreactivity.

### Double staining of caspase-12 and TUNEL

Double staining of caspase-12 and the TUNEL was performed using the In Situ Cell Death Detection Kit (Roche Diagnostics). After immunohistochemical staining of capase-12 as described above, the sections were washed three times in PBS and then incubated with the TUNEL solution containing FITC-dUTP for 60 min at 37°C. Finally, the sections were washed in PBS and mounted for the analysis. All sections were examined by a Nikon confocal microscope (Nikon Corporation, Tokyo, Japan) equipped with a Nikon color video camera. The images were adjusted using Adobe Photoshop CS (8.0) software (Adobe Systems, Mountain View, CA, USA). Then, we counted the TUNEL or Caspase 12 positive SGNs basing on the morphology and size of the SGNs.

### Western blot analyses

For Western blotting, the midmodiolar tissue was gathered according to the method of Szabo’ et al. and Bae et al [14,16]. After decapitated, the temporal bones were separated from the skull, and the spiral lamina from the rest of the bony cochlea was removed delicately. Following removal spiral lamina, more and more modiolus was exposed and sticked out of the temporal bone. Then, the modiolus could be easily separated from the temporal bone. When the modiolus removal was completed, it was broken up into several small pieces by using a delicate pair of forceps. Extracted midmodiolar tissues were lysed in the solution consisting of 50 mM Tris (pH7.4), 150 nM NaCl, 1% Triton X-100, 1% sodium deoxycholate, 0.1% SDS and protease inhibitor cocktail. Lysates were ultrasonicated, followed by immediately denatured for 10 min at 100°C. Total cell lysates were electrophoresed in 10% SDS-PAGE. After wet transfer to PVDF membranes and incubation in blocking buffer (5% nonfat dry milk in TBS supplemented with 0.1% Tween 20), the membranes were incubated with the primary antibody overnight at 4°C.The following antibodies were used: anti-caspase-12 (1:500), anti-CHOP (1:500), anti-Bip (1:500), anti-ATF-6α (1:200), anti-IRE1α (1:200), anti-Phospho-eIF2-alpha (Ser51) (1:200), anti-Phospho-PERK (Thr980) (1:200), and anti-α-tubulin (1:1000). After three washes in TBST, membranes were incubated with peroxidase-conjugated secondary antibodies (1:3000) for 1 h at room temperature and subsequently washed as described earlier. Detection was performed by chemiluminescence using an Enhanced Chemiluminescence kit and exposed to Kodak X-omat BT film (Kodak, USA). Strip off the primary and secondary antibodies with a stripping buffer. The immunoreactive bands were processed as above with a different primary antibody. Each western blot experiment was performed at least three times with similar results. The band density was quantified using Quantity One image processing program (Bio-Rad, USA) and normalized to that of the control group.

### Statistical analyses

Statistical analysis was performed by one way ANOVA of variance analysis using SPSS 18.0 software (SPSS Inc. Chicago, IL, USA). All values are represented as mean ± SEM. LSD multiple comparison test was used to evaluate the effect among groups. In all analyses, the value of *P*≤0.05 was considered to be significant.

## Results

### Auditory function

**Figure 1:**
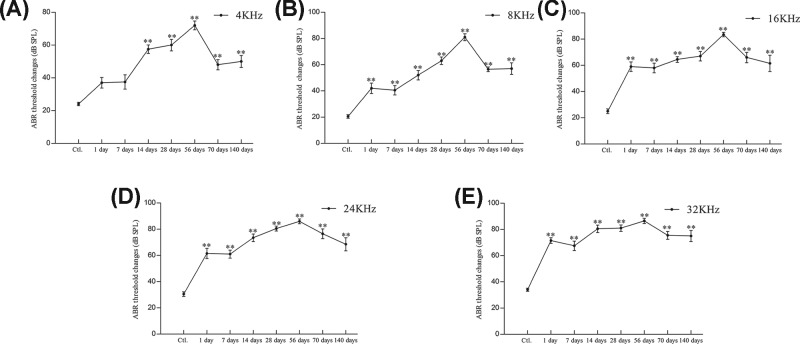
Auditory function Tone-burst ABR threshold changes at 4, 8, 16, 24, and 32 KHz frequencies at each time point after kanamycin treatment (panels (**A**–**E**) represent 4, 8,16, 24, and 32 KHz, respectively). Significance: **P<0.01 compared with control; Ctl, Control.

In the control group, the tone burst ABR average thresholds at 4, 8, 16, 24, and 32 KHz frequency were 24.00 ± 3.16, 20.50 ± 4.38, 25.00 ± 5.77, 30.50 ± 5.50, and 34.00 ± 3.94 dB sound pressure level (SPL), respectively. At 1, 7, 14, 28, 56, 70, and 140 days after kanamycin treatment, the tone burst ABR average thresholds at 4KHz were 37.00 ± 10.33, 37.50 ± 13.79, 57.00 ± 8.24, 60.00 ± 11.06, 72.00 ± 8.56, 48.00 ± 9.78, and 50.00 ± 11.55 dB SPL, respectively (Figure 1A). At each time-point after kanamycin treatment as above, the tone burst ABR average thresholds at 8KHz were 42.00 ± 12.51, 40.50 ± 11.17, 52.00 ± 11.35, 63.00 ± 9.19, 81.00 ± 8.43, 56.50 ± 5.80, and 57.00 ± 14.18 dB SPL, respectively (Figure 1B). At 16KHz frequency, the average thresholds were 59.00 ± 11.50, 58.00 ± 11.83, 64.50 ± 6.85, 67.00 ± 11.35, 83.50 ± 5.30, 66.00 ± 12.43, and 61.50 ± 19.59 dB SPL, respectively (Figure 1C). At 24KHz frequency, the average thresholds were 61.50 ± 12.26, 61.00 ± 9.66, 73.50 ± 9.14, 80.50 ± 5.99, 86.00 ± 5.68, 76.50 ± 11.80, and 68.50 ± 15.64 dB SPL, respectively (Figure 1D). At 32KHz frequency, the average thresholds were 71.50 ± 6.69, 67.50 ± 11.37, 80.50 ± 8.96, 81.00 ± 7.75, 86.50 ± 6.26, 75.50 ± 9.56, and 75.00 ± 13.33 dB SPL, respectively (Figure 1E). These results indicate that the tone burst ABR average thresholds of each frequency at each time-point were significantly increased after kanamycin treatment (*P*<0.001, compared with control group, Figure 1A–E). In addition, the most severe hearing impairment was observed 56 days after kanamycin treatment at 24 and 32 KHz with a hearing loss of up to 50 dB SPL.

### Time-course of rat SGN loss after kanamycin treatment

**Figure 2:**
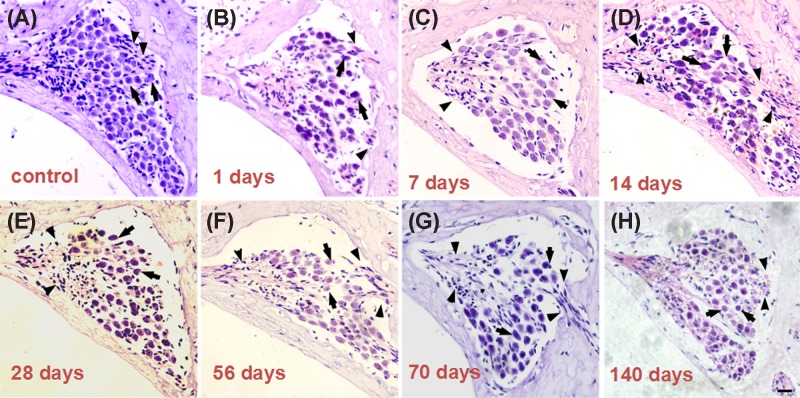
Time-course of rat SGN loss after kanamycin treatment Light micrographs of Rosenthal’s canal in the basal turn from the eight different groups ((**A**) represents control group; panels (**B**–**H**) represent 1, 7, 14, 28, 56, 70 and 140 days after kanamycin treatment, respectively). (A–H) shows SGCs (arrowheads) and nerve fibers (arrow) in the spiral ganglion; scale bar = 20 μm.

**Figure 3:**
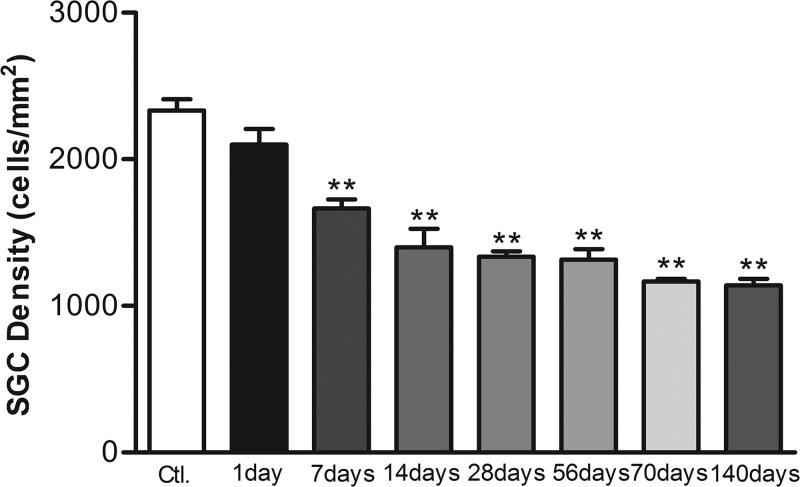
SGC densities changes after kanamycin treatment Mean SGC densities changes in the basal turn of the cochlea in the experimental groups; **P<0.01. Statistical analyses were carried out between the control and each experimental group. Error bars stand for S.E.M.

Figure 2 shows light micrographs of the Rosenthal’s canal in the basal turn of the cochlea and provide representative examples of the eight different groups of cochleae (the control group and at 1, 7, 14, 28, 56, 70, and 140 days after kanamycin treatment). In the control group, the Rosenthal’s canal was full of type I and/or type II SGCs. At 1 day after kanamycin administration, the cellular distribution of SGCs within the spiral ganglia appeared unchanged (Figure 2B). A dramatic loss of SGCs could be observed at 7 days after deafening, which became more significant at 14, 28, 56, 70, and 140 days after deafening (Figures 2 and 3). There were significant differences in SGC counts between the control group and each experimental group at all observed time-points except for the first day after kanamycin treatment. SGN densities were obviously reduced at days 7 and 14 after kanamycin treatment.(*P*<0.01, Figure 3).

### Ultrastructural variation of SCG after kanamycin treatment

**Figure 4:**
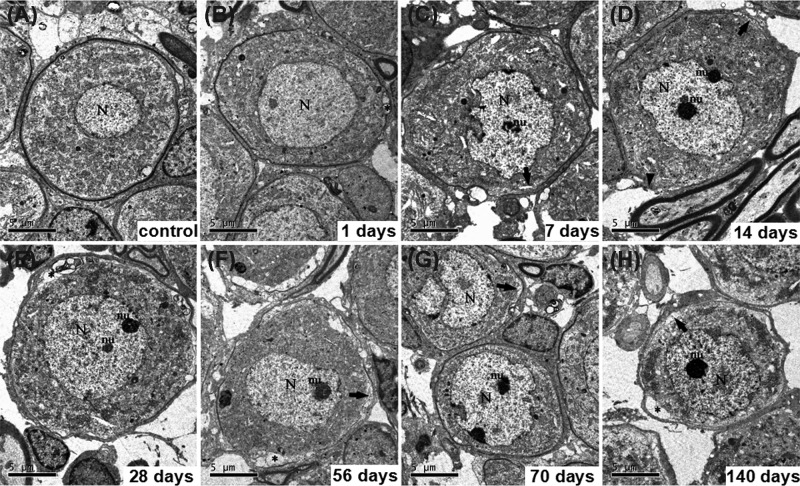
Ultrastructural variation of SCG after kanamycin treatment Ultrastructural features of SGNs after kamamycin treatment (panels (**B**–**H**), represent 1, 7, 14, 28, 56, 70 and 140 days after kanamycin treatment, respectively). (**A**) shows SGCs from a normal cochlea. A vacuole was found between the neuron and satellite cell (asterisk, (B, E, F and H)). Perikaryal myelin of the atypical type I neurons became looser (arrow, (C, D, F, G and H)). Arrowhead indicates loss of myelin sheath (D). N, nucleus; nu, nucleolus; scale bar = 5 μm.

**Figure 5:**
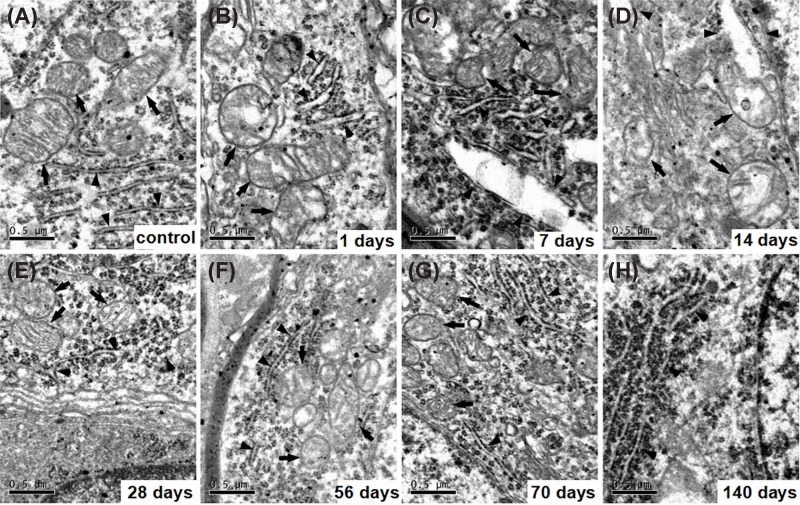
Electron micrographs of morphological structures at the subcellular level of representative SGCs Electron micrographs of morphological structures at the subcellular level of representative SGCs from the eight different groups were detected (panels (**B**–**H**), represent 1, 7, 14, 28, 56, 70 and 140 days after kanamycin treatment respectively). (**A**) shows ER (arrowheads) and mitochondria (arrows) in SGCs from a normal cochlea. The endoplasmic reticula were dilated obviously (arrowheads, (B–D)) and the mitochondria were progressively swelling (arrows, (B–D)), and gradually recovered after kanamycin treatment (arrows and arrowheads, (E–H)). Meanwhile, focal vacuoles in mitochondria occurred (arrows, (B–F)); scale bar = 0.5 μm.

In the control group, SGCs showed a prominent myelin sheath formed by the enveloping Schwann cell and which had an ovoid shape (Figure 4A). The perikaryon contained a large, round, and relatively electron-lucent nucleus with one or more prominent nucleoli. The cytoplasm contained large aggregates of cisternae of the rough endoplasmic reticula and stacks of the Golgi apparatus, as well as large numbers of mitochondria (Figure 5A). At 1 day after kanamycin administration, most SGCs exhibited a cell shape and ultrastructural appearance that were similar to their counterparts in the control cochlea (Figure 4B). At 7, 14, 28, 56, 70, and 140 days after kanamycin treatment, the amount of type I SGCs had dramatically diminished, resulting in an enlargement of the free space between the individual cells (Figure 4C–H). The SGCs exhibited a different shape from the normal type I SGCs. Although some cells retained an ovoid shape, most cells appeared dendritic. Most of the SGCs exhibited a more compact distribution of organelles and intracellular content, resulting in an electron-dense appearance. Meanwhile, their perikaryal myelin became thinner and looser. Figure 5 shows electron micrographs (×28,500) of morphological structures at the subcellular level of representative SGCs from the eight different groups. SGCs from the control group contained normal morphological endoplasmic reticula and mitochondria (Figure 5A), whereas the endoplasmic reticula in the kanamycin-treatment groups were dilated and the mitochondria were swollen at 1 day after kanamycin treatment (Figure 5B–D). The effect on the ER and the mitochondria progressed until day 14 day and then gradually recovered from 28 to 140 days after kanamycin treatment (Figure 5E–H). At 140 days after kanamycin treatment, the focal vacuoles in the mitochondria of SGNs remained, whereas the dilation of the ER was generally recovered.

### Caspase-12 expression in SGCs and TUNEL staining

**Figure 6:**
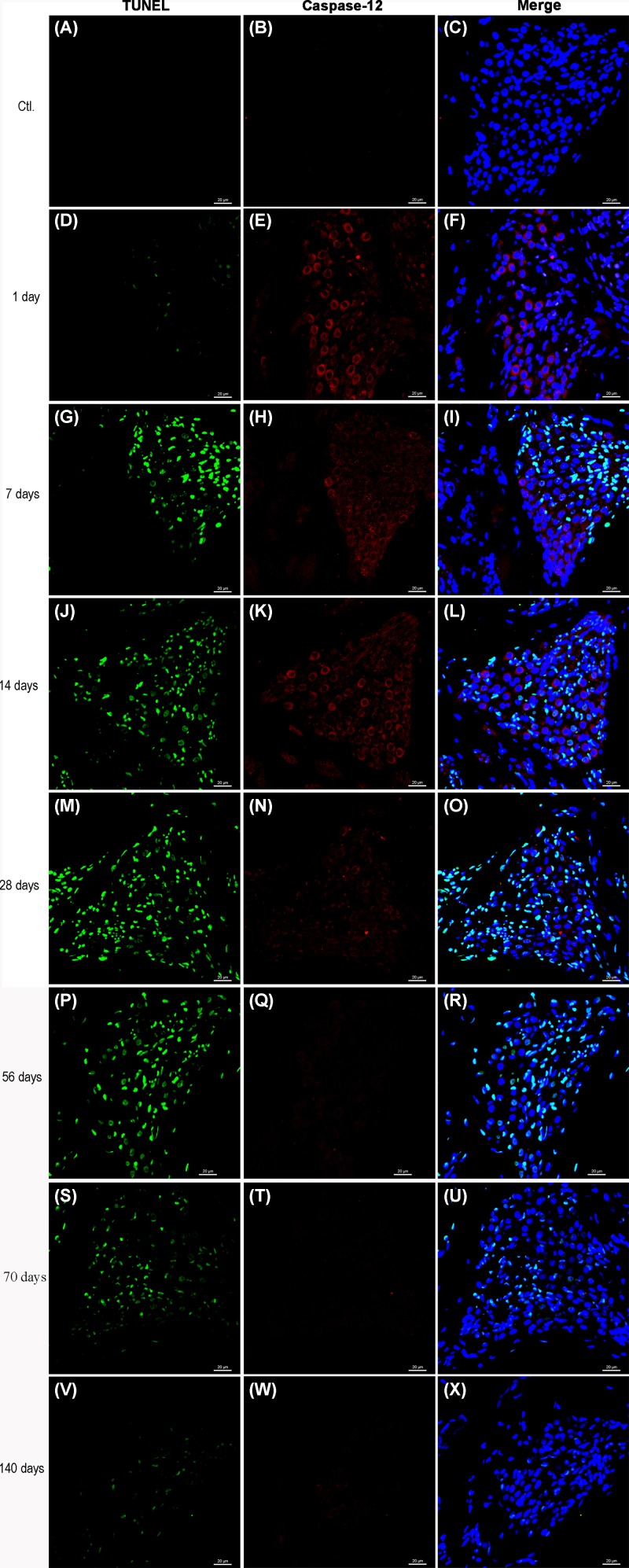
Caspase-12 expression in SGCs and TUNEL staining Double staining of caspase-12 (red) and TUNEL (green) of the basal turn of cochleae at 0 day (**A**–**C**), 1 day (**D**–**F**), 7 days (**G**–**I**), 14 days (**J**–**L**), 28 days (**M**–**O**), 56 days (**P**–**R**), 70 days (**S**–**U**) and 140 days (**V**-**X**). The expression of caspase-12 was observed in part of TUNEL positive cells. Scale bar = 20 μm.

Double-labeled immunofluorescent staining was performed at different times after kanamycin treatment to examine caspase-12 expression and DNA fragmentation (TUNEL) at the cellular level. As shown in Figure 6, the expression of caspase-12 in SGCs was increased at 1, 7, 14, and 28 days after kanamycin treatment in comparison with the control group (Figure 6E, H, K, and N), while the number of TUNEL-positive SGCs was increased at 7, 14, 28, 56, 70, and 140 days after kanamycin treatment (Figure 6G, J, M, P, S, and V). At 7, 14, and 28 days after kanamycin treatment, the number of SGCs that was positive for both TUNEL and caspase-12 was increased. As shown in Supplementary Figure S1, TUNEL positive cells peaked at 28 days. But expression of Caspase- 12 reach peak at 7 days, suggesting other apoptotic pathway may be involved in this process. Not all the TUNEL-positive cells exhibited immunoreactivity to caspase-12. In addition, the caspase-12-positive SGCs were not all TUNEL-positive.

### ER stress is involved in the kanamycin treatment-induced SGN apoptosis

**Figure 7:**
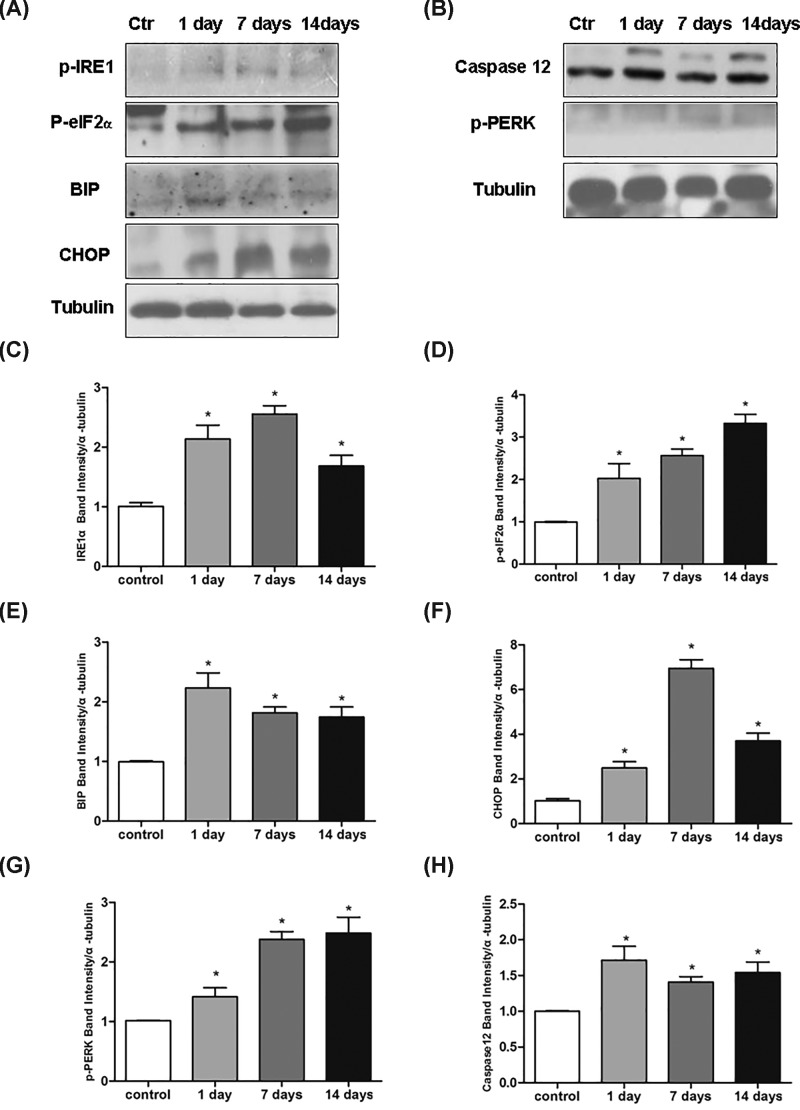
ER stress is involved in the kanamycin treatment-induced SGN apoptosis ER stress is involved in the kanamycin treatment-induced SGN apoptosis. The expression level of p-IRE1α, p-eIF2α, Bip, Chop, p-PERK, and caspase-12 were detected after kanamycin treatment (**A–H**). Data were shown as the mean ± S.E.M.; *P<0.05 as compared with control. *n*=3 for each protein examined.

To investigate the roles of ER stress in the kanamycin treatment-induced SGN apoptosis, we measured the expression of phospho-PERK (Thr980), phospho-eIF2-α (Ser51), IRE1α, ATF-6α, Bip, CHOP, and caspase-12 after kanamycin treatment by Western blotting. The expression of p-PERK, p-eIF2α, p-IRE1α, Bip, caspase-12, and Chop was significantly unregulated after kanamycin treatment (Figure 7). However, the expression of ATF-6α was hardly detected in the control and experimental groups (data not shown). These results indicated that kanamycin treatment induced ER stress in SGNs.

## Discussion

Due to the side effects of kanamycin that limits its clinical application, many researchers explore the mechanism of aminoglycoside ototoxicity. In this study, we examined the dynamic morphological changes of SGCs after kanamycin treatment and investigated the possible mechanism involved in the degeneration of SGNs. A progressive loss of SGNs was found at day 14 after kanamycin treatment with the number of SGCs declining to ∼40% of the normal population. At day 70 after kanamycin administration, the value described above reached ∼50%. The SGNs death is a consequence of reduced neurotrophic support from hair cells at early stage. However, supporting cells remaining in the sensory epithelium after hair cell loss provide neurotrophic support to SGNs. Neurotrophic support is insufficient to rescue the SGNs in the long-term, but it may slow their death and and their transit through the apoptotic process. This degeneration process was comparable to the study of Tan and Shepherd, who treated adult rats with gentamicin sulfate and frusemide [17]. Bichler et al. found that after deafening by systemic amikacin treatment from days 10 to 26 of age, 85–90% SGNs of the rat disappear following the complete destruction of the organ of Corti, and 10–15% of those remaining were atypical type I or type II neurons [4]. These atypical type I neurons are generally large and have abundant cisternae of endoplasmic ER, yet lack a compact myelin sheath, and are named type III neurons accordingly. Type I spiral ganglion cells comprise the vast majority of spiral ganglion cells (90–95% in cats and 88% in humans), and exclusively innervate the inner hair cells. They are myelinated, bipolar neurons. Type II spiral ganglion cells make up the remainder [18]. Type II spiral ganglion cells are unipolar and unmyelinated in most mammals. They innervate the outer hair cells, with each Type II neuron sampling many outer hair cells. Type III neurons resemble type I neurons except for being unmyelinated. Type III neurons include plentiful cytoplasmic organelles, satellite cells on their processes surrounding the soma, and the absence of a myelin sheath, which resemble postnatal developing SGCs that have been previously observed in other studies [19].

Our TEM investigations revealed that most SGCs were generally dendritic in shape and exhibited a more compact distribution of organelles and other intracellular content. Meanwhile, their perikaryal myelin became thinner and looser. The structural analysis data is analogous to the study of Dodson et al. and further support that hair cell loss induces reversion of mature SGNs to a less differentiated form [20]. However, the rate of SGN loss that we observed was different to that reported by Bichler et al. on rats deafened by systemic amikacin treatment and Alam et al. on rats deafened by systemic kanamycin treatment [4,5]. These differences are possibly due to these studies being performed largely in the developing auditory systems, whereas our study focused on the mature auditory system. In adult rats, the deafferented SGNs retain their projection to the cochlear nucleus, a putative source of neurotrophic support, and receive neurotrophic support from Schwann cells and supporting cells [5]. Therefore, deafferentation is likely to deprive only a fraction of the neurotrophic support available to SGNs.

**Figure 8:**
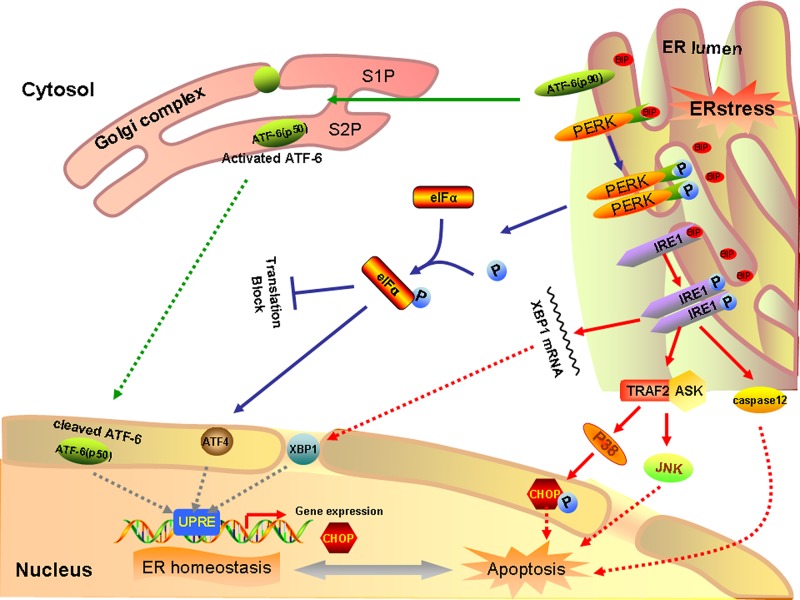
A schematic of unfolded protein response signaling pathway In response to ER stress, Bip dissociates from ER stress transducers and binds to unfolded and misfolded proteins, resulting in the activation of ER stress transducers PERK, IRE1 and ATF6. Upon activation, PERK increases phosphorylation of eIF2α, leading to a global attenuation of protein synthesis and a concomitant increase in ATF4 translation. In turn, ATF4 induces CHOP, a proapoptotic transcription factor. After the dissociation of Bip, IRE1 splices the mRNA of XBP1, and produces an active transcription factor named spliced XBP1, which upregulates ER chaperones and proteins implicated in the ER-associated protein degradation. Furthermore, IRE1 recruits TRAF2 to trigger activation of ASK1 and JNK resulting in SGC apoptosis. Meanwhile, TRAF2 liberates procaspase 12 and allows its dimerization and translocation in the cytosol, finally leading to apoptosis. Besides, ASK1 can also activate p38, the activated state of which functions to phosphorylate CHOP, thereby enhancing its pro-apoptotic effect. In addition, ATF6 translocates to Golgi apparatus, where it is activated by site 1 and site 2 Golgi resident proteases. Activated ATF6 transcriptionally induces ERAD genes and upregulates CHOP expression.

The death of the deafferented SGNs appears to be via apoptosis. Although death receptor- and mitochondria-mediated SGN apoptosis have been studied extensively after aminoglycoside treatment [14,21], only a few studies have indicated that aminoglycoside treatment could directly induce ER stress in SGNs. PERK, ATF6, and IRE1 signaling pathways can trigger pro-apoptotic signals during chronic ER stress through the activation of downstream molecules, such as CHOP for PERK and ATF6 or JNK for IRE1 (Figure 8). Caspase-12, which is localized specifically on the ER, is involved in ER-specific apoptosis independently of mitochondria or death receptors. In our study, at the early stage of post kanamycin treatment, the ER of SGNs was dilated and the levels of Bip, IRE1α, p-PERK, p-eIF2α, CHOP, and caspase-12 was significantly upregulated. These results indicate that ER stress was induced in the SGNs after kanamycin treatment through the PERK and IRE1 pathways. However, we did not find any upregulation of the ATF6 after kanamycin treatment, suggesting ATF6 pathway is not involved in kanamycin-induced ER stress. As shown in Figure S1, TUNEL positive cells peaked at 28 days. But expression of Caspase12 reach peak at 7 days, suggesting other apoptotic pathway may be involved in this process. Indeed, there are other apoptotic pathway may be involved in this process. Recent study has reported that the expression levels of E2F1 and cyclin dependent kinase 1 (CDK1) were obviously up-regulated at 1 and 3 days after kanamycin treatment. Cleaved caspase-9 also increased robustly 1 or 2 weeks after the deafening procedure. These results suggested that the E2F1–CDK1 pathway represented an important role in kanamycin-induced spiral ganglion cell apoptosis [22]. Kanamycin treatment can induce ER stress, possibly due to its antibacterial mechanism, which can interfere with microbial ribosomes and result in the formation of reactive oxygen species (ROS) [23]. Jeong et al. found that SGC apoptosis is mediated by ROS and the JNK signaling pathway after gentamicin treatment [24]. Recently, the relationship between ER stress and ROS has attracted much interest [25,26]. Overexpression of CHOP has been reported to result in apoptosis, while CHOP^−/−^ mice exhibit reduced apoptosis in response to ER stress [27]. Thus, caspase-12 and CHOP might take part in promoting ER stress-induced SGN apoptosis after kanamycin treatment. These results indicated that kanamycin treatment might induce SGN apoptosis through activated ER stress.

In conclusion, we have demonstrated for the first time that ER stress was involved in kanamycin-induced apoptosis of SGNs. Kanamycin treatment-induced SGN apoptosis is mediated, at least in part, by ER stress-induced upregulation of caspase-12 and CHOP. Because functional SGNs are essential for the success of restoring hearing function, such as after cochlear implants to ameliorate deafness, understanding these neurodegenerative events at the molecular level may identify ER stress as a promising drug target to alleviate secondary SGN degeneration in deafness.

## Supporting information

**Supplementary Figure 1:** 

## Abbreviations

ABRs: evoked auditory brain stem responses
ASK1: apoptosis signal regulating kinase 1
ATF6: activating transcription factor 6
CDK1: cyclin dependent kinase 1
CHOP: C/EBP Homologous Protein
dUTP: 2′-deoxyuridine 5′-triphosphate
ER: endoplasmic reticulum
ERAD: Endoplasmic Reticulum Associated Degradation
IRE1: inositol requirement 1
JNK: c-Jun N-terminal kinase
LSD: Least significant difference
PERK: interferon-induced double stranded RNA-activated protein kinase (PRKR) -like endoplasmic reticulum kinase
ROS: reactive oxygen species
SGCs: spiral ganglion cells
SGN: spiral ganglion neuron
SNHL: severe sensorineural hearing loss
SPL: sound pressure level
TEM: transmission electron microscopy
TUNEL: terminal deoxynucleotidyl transferase-mediated dUTP nick end labeling
UPR: unfolded protein response.
