# Supplementary figures and images for: Endoplasmic reticulum stress is involved in spiral ganglion neuron apoptosis following chronic kanamycin-induced deafness
Source: Biosci Rep. 2019 Feb 8;39(2):BSR20181749. doi: 10.1042/BSR20181749 (PMC6592474; doi:10.1042/BSR20181749)

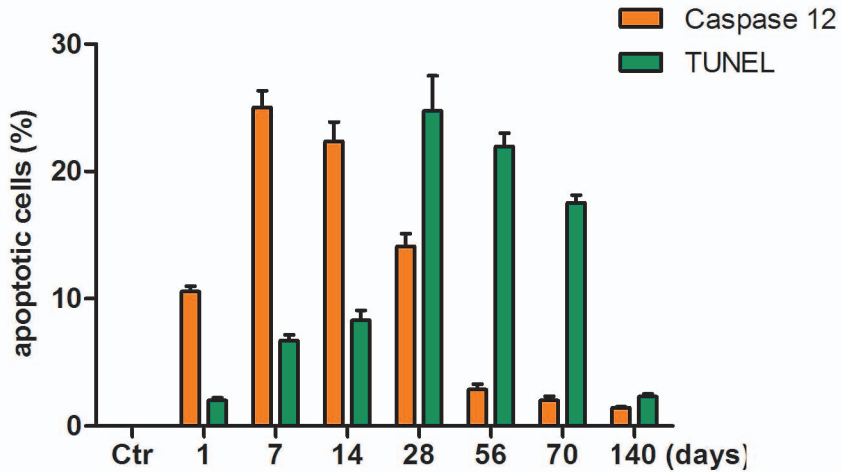

Supplement: Supplementary file 1 [file bsr-39-bsr20181749_Supp1.pdf]
